# Prognosis of severe drug-induced acute interstitial nephritis requiring renal replacement therapy

**DOI:** 10.1080/0886022X.2021.1942914

**Published:** 2021-06-30

**Authors:** Li Huang, Shaoshan Liang, Jianhua Dong, Wenjing Fan, Caihong Zeng, Ti Zhang, Shuiqin Cheng, Yongchun Ge

**Affiliations:** National Clinical Research Center of Kidney Diseases, Jinling Hospital, Nanjing University School of Medicine, Nanjing, China

**Keywords:** Drug, acute interstitial nephritis, renal replacement therapy, prognosis

## Abstract

**Objective:**

Drug-induced acute interstitial nephritis (DAIN) is often associated with improved outcomes, whereas some patients may still progress to chronic kidney disease (CKD). The aim of this study was to evaluate the prognosis of patients with severe DAIN requiring renal replacement therapy (RRT) at baseline, and to explore the risk factors of progression to CKD.

**Methods:**

We performed a retrospective study of patients with severe DAIN confirmed by renal biopsies in our center over a 10 years period, all the patients received RRT at presentation. The clinical and pathological characteristics at baseline were recorded, and the outcomes (renal function recovered or progressed to CKD) during follow-ups were also evaluated. Univariate and multivariate logistic regression analysis were performed to identify the independent risk factors of progression to CKD.

**Results:**

Seventy-two patients who met the inclusion criteria were enrolled, 13 patients (18.0%) progressed to CKD (GFR < 60 ml/min/1.73 m^2^) after at least 6 months of follow-up, the remaining 59 patients achieved a favorable renal function recovery. Compared with patients who achieved renal function recovery (recovery group), the patients progressed to CKD (progression group) were older and had longer interval from symptom onset to treatment with steroids. The peak serum cystatin C concentration was higher in progression group than recovery group. Higher score of interstitial fibrosis/tubular atrophy (IFTA) and more interstitial inflammatory cells infiltration were detected in renal tissue in progression group. According to multivariable analysis, higher peak cystatin C concentration (OR = 2.443, 95% CI 1.257, 4.746, *p* = 0.008), longer interval to treatment with corticosteroids (OR = 1.183, 95% CI 1.035, 1.352, *p* = 0.014) were independent risk factors of progression to CKD. The cutoff value of cystatin C concentration was 4.34 mg/L, at which the sensitivity and specificity were 76.9% and 89.3%, respectively; the cutoff value of interval to treatment with corticosteroids was 22.5 days, at which the sensitivity and specificity were 81.8% and 79.5%, respectively.

**Conclusion:**

Renal function was reversible in majority of patients with severe DAIN requiring RRT when early identification and treatment. Higher peak cystatin C concentration and longer interval to treatment with corticosteroids associated with worse renal prognosis.

Acute interstitial nephritis (AIN) is an increasingly recognized cause of acute kidney injury (AKI). It is pathologically characterized by inflammation and edema of the renal interstitium and tubulitis, sparing the glomeruli and the vasculature. The true incidence was unknown and probably a large number of cases with mild renal dysfunction were remained unrecognized. Ongoing inflammation associated with unrecognized and untreated AIN leads to kidney fibrosis, and 40–60% of patients with AIN ultimately progress to chronic kidney disease (CKD) [1–3].

The etiology of AIN can be classified as drugs, infections, autoimmune, systemic diseases, or idiopathic. Drugs were the major cause of AIN over the past 2–3 decades. There has been a changing profile of associated drugs, it changed from the 1970s and 1980s when most cases were penicillin-related and associated with typical hypersensitivity symptoms to recent decades when etiology became more diverse and there are less associated symptoms [4]; antibiotics, non-steroidal anti-inflammatory drugs (NSAIDs) and proton pump inhibitors (PPIs) are the most commonly implicated drug categories as reported [5,6]. Although drug-induced AIN (DAIN) has been reported to have increased likelihood of complete recovery [7,8], the published data on long-term prognosis, especially in patients with severe AIN (i.e., requiring renal replacement therapy) are lacking.

In the present study, we retrospectively evaluated the renal outcomes of patients with severe DAIN who received renal replacement therapy (RRT) at baseline during the past 10 years and further identified the risk factors associated with progression to CKD.

## Methods

### Study patients

In this retrospective study, we reviewed the records of patients who underwent native renal biopsy between 2009 and 2019 at the National Clinical Research Center of Kidney Diseases, Jinling Hospital, Nanjing University School of Medicine. There were 2433 patients with renal biopsy confirmed AIN were screened. All of the patients received renal ultrasonography examination and showed normal or relatively large renal size before biopsy. The inclusion criteria of this study were patients > 14 years of age, with a definitely medication history before AKI onset, received RRT after admission, and had a minimum of 6 months follow-up. The exclusion criteria were patients consistent with autoimmune or malignant diseases, chronic or acute glomerulonephritis, AIN with uveitis syndrome (TINU), already initiated RRT or corticosteroids therapy before admission. Whether the patients received corticosteroids, the dosage and route of steroids administration were decided by the physician. This study was reviewed by the Ethics Committee of Jinling Hospital (2014KLY-001).

Age, gender, history of hypertension or diabetes mellitus, causative agents, 24-h urinary protein excretion, urinary sediment blood cell counts, urinary *N*-acetyl-β-d-glucosaminidase (NAG), urinary retinal-binding protein (RBP), hemoglobin, leukocyte, peak creatinine, peak cystatin C, renal ultrasonography, length of RRT-dependence time, and dosage of corticosteroids were recorded. Intervals from symptoms onset to treatment with corticosteroids and duration for RRT were also reviewed. The estimated glomerular filtration rate (eGFR) was calculated by the Chronic Kidney Disease Epidemiology Collaboration (CKD-EPI) equation and expressed as milliliters per minute per 1.73 m^2^.

### Pathological characteristics

The tissue for light microscopy was serially sectioned and stained using hematoxylin and eosin, periodic acid-Schiff, methenamine-silver, and Masson trichrome. Cryosections were stained with fluorescein isothiocyanate-conjugated rabbit anti-human IgG, IgA, IgM, C3, C1q, kappa, and lambda. The tissue for electron microscopy was processed according to standard protocols. Interstitial inflammatory cell infiltration, acute tubular injury (ATI), and interstitial fibrosis/tubular atrophy (IFTA) were scored semi-quantitatively based on the percentage of the tubulointerstitial compartment and recorded as 0% (negative), 1–25% (mild), 26–50% (moderate), or >50% (severe). All biopsy slides were re-reviewed by renal pathologist (SL) for quantifying the lesions.

### Renal outcomes during follow-ups

All the patients had scheduled follow-up of at least 6 months from the date of renal biopsy. Serum creatinine, urinalysis and eGFR were recorded to evaluate the renal outcomes. The CKD was defined as abnormalities of kidney function (GFR < 60 mL/min/1.73 m^2^) or albuminuria or urine sediment abnormalities, present for more than 3 months. The renal recovery was defined as eGFR ≥ 60 mL/min/1.73 m^2^ without albuminuria or hematuria during 6 months of follow-up.

### Statistical analysis

Continuous variables and normally distributed data were expressed as the mean ± standard deviation (SD) and differences between groups were analyzed using Student’s *t*-test. Non-normally distributed data were expressed as median and interquartile range and differences between groups were analyzed using the rank-sum test. For non-continuous variables, frequency distribution was used and were compared using the Chi-squared test. A multivariable logistic regression was used to identify independent risk factors of progression to CKD. Results are expressed as odd ratio (OR) with 95% confidence interval (95% CI). Kaplan–Meier survival curves were used to compare the probability of renal recovery between groups with different peak serum cystatin C levels and interval to corticosteroids treatment, and the curves were compared using a log-rank test. Statistical significance was defined as *p*-values < 0.05. Analyses were performed using the SPSS 19.0 Statistics software (SPSS, Chicago, IL).

## Results

### General clinical information

Seventy-two patients (40 males and 32 females) with a mean age of 47 ± 14 years old and a confirmed diagnose of DAIN were enrolled in the present study, all of them received prolonged intermittent renal replacement therapy (PIRRT) after admission. During the 6–130 months of follow-up, all the patients weaned from dialysis eventually, 59 patients achieved complete recovery of renal function with no albuminuria or hematuria at 6 months, and the remaining 13 patients progressed to CKD. We compared the clinical and pathological characteristics of patients who recovered completely (recovery group) and those who progressed to CKD (progression group) at the end of the 6th month during follow-ups. The baseline clinical and demographic characteristics of patients are summarized in Table 1. There was no significant difference in gender, comorbid hypertension, diabetes, length of RRT-dependence time between the two groups (*p* > 0.05). Compared with patients in recovery group, the patients in progression group were older (55 ± 9 versus 45 ± 14 years, *p* = 0.016) and had longer interval from symptom onset to treatment with corticosteroids (32.5 ± 20.1 versus 13.1 ± 7.7 days, *p* = 0.001). There was no significant difference in laboratory characteristics including leukocyte, hemoglobin, and peak creatinine (*p* > 0.05) except serum peak cystatin C concentration (5.58 ± 1.87 versus 3.50 ± 1.22 mg/L, *p* < 0.001). There was no significant difference in proteinuria, hematuria, leukocyturia, and urinary NAG (*p* > 0.05) except urinary RBP concentration (*p* < 0.001).

**Table 1.** Clinical characteristics of patients with severe DAIN at baseline.

	Total (*n* = 72)	Recovery group (*n* = 59)	Progression group (*n* = 13)	*p* Value
Age (years)	47 ± 14^a^	45 ± 14	55 ± 9	0.016
Gender (M/F)	40/32	35/24	5/8	0.171
Hypertension, *n* (%)	17 (23.6%)	15 (25.4%)	2 (15.4%)	0.681
Diabetes, *n* (%)	11 (15.2%)	11 (18.6 %)	0	0.206
Interval to treatment with steroids (days)	16.8 ± 12.8	13.1 ± 7.7	33.1 ± 17.4	0.001
Length of RRT- dependence (days)	10 (6,12) ^b^	10 (6,12)	8 (5.5,13)	0.930
Hemoglobin (g/l)	114 ± 20	115 ± 19	109 ± 23	0.309
Leukocyte (10^9^/l)	8.00 (6.41,11.10)	8.05 (6.31,11,1)	7.30 (6.46,11.72)	0.830
Peak creatinine (mg/dl)	10.1 ± 3.0	10.1 ± 2.8	9.7 ± 3.8	0.677
Peak cystatin C (mg/l)	3.92 ± 1.59	3.50 ± 1.22	5.58 ± 1.87	<0.001
Proteinuria (g/24h)	0.43 (0.24,0.74)	0.41(0.21,0.69)	0.73 (0.27,1.30)	0.104
Hematuria, *n* (%)	26 (36.6%)	23 (38.3%)	3 (27.3%)	0.735
Leucocyturia, *n* (%)	14 (19.7%)	12 (20.0%)	2 (18.2%)	1.000
Urinary NAG (U/g*cr)	23.1 (16.1,42.8)	21.7 (14.0, 42.2)	32.5 (20.2,45.4)	0.142
Urinary RBP (mg/l)	9.9 (2.1,21.9)	6.6 (2.0, 18.8)	25.2 (8.5, 42.0)	0.002

RRT: renal replacement therapy; NAG: N-acetyl-β-d-glucosaminidase; RBP: retinal-binding protein.

^a^Mean ± SD.

^b^Median (interquartile range).

**Figure 1.**
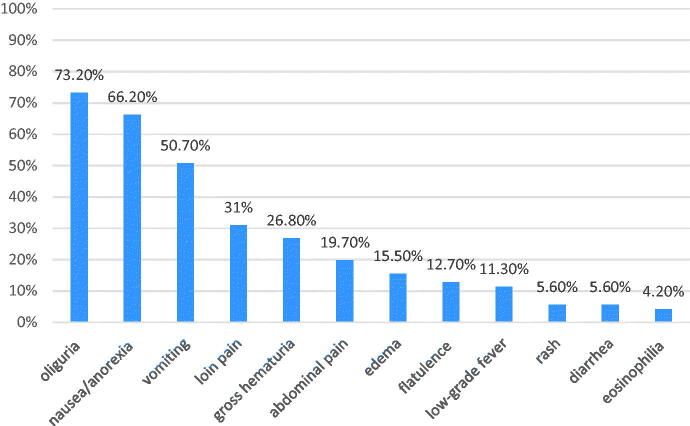
Occurrence of symptoms (%) in the patients with severe DAIN at presentation.

All the patients have symptoms in the period of drugs exposure except one patient, whose renal dysfunction was detected because of melena and gastrointestinal bleeding. The presenting symptoms included oliguria occurred in 52 (73.2%) patients, nausea/anorexia in 47 (66.2%) patients, vomiting in 36 (50.7%) patients, loin pain in 22 (31%) patients, gross hematuria in 19 (26.8%) patients, abdominal pain in 14 (19.7%) patients, edema in 11 (15.5%) patients, flatulence in 9 (12.7%) patients, low-grade fever in 8 (11.3%) patients, rash in 4 (5.6%) patients, diarrhea in 4 (5.6%) patients, and eosinophilia in 3 (4.2%) patients (Figure 1). None of the patient has the triad of low-grade fever, rash, and eosinophilia concurrently.

### Causative agents of DAIN

Majority (*n* = 62, 86.1%) of patients had multiple drugs exposure, antibiotics (86.1%) were prescribed in 62 patients while the most common types of cephalosporins (*n* = 24, 33.3%), clindamycin (*n* = 16, 19.2%), quinolones (*n* = 14, 19.4%), and penicillins (*n* = 12, 16.6%). NSAIDs were prescribed in 16 (22.2%) patients, PPI were prescribed in 6 (8.3%) patients. Four patients received traditional Chinese herbs. None of these drugs was prescribed in our hospital, so accurate exposure time and dose could not be obtained.

### Pathologic characteristics

Global glomerulosclerosis was observed in 46 (63.9%) patients. ATI was scored as mild in 7 (9.7%) patients, moderate in 45 (62.5%) patients, and severe in 20 (27.8%) patients. IFTA was scored as mild in 19 (26.3%) patients, moderate in 7 (9.7%) patients, and severe in 1 (1.4%) patient. Interstitial inflammatory cells infiltration was scored as mild in 21 (29.2%) patients, moderate in 33 (45.8%) patients, and severe in 18 (25.0%) patients. Arterial intimal thickening was present in 39 (54.2%) patients. Staining of IgG, IgA, IgM, C3, C1q, kappa, and lambda were all negative. The histologically profiles of patients in two groups are shown in Table 2. Patients who progressed to CKD had higher score of IFTA (*p* < 0.001) and more interstitial inflammatory cells infiltration (*p* = 0.002). There was no significant difference of lymphoplasmacytic, eosinophils, and mononuclear cells infiltration between two groups. Interstitial fibrosis was correlated with interval to treatment with corticosteroids (*r* = 0.532, *p* < 0.01), proteinuria (*r* = 0.281, *p* = 0.020), hemoglobin (*r* = −0.293, *p* = 0.013), and peak cystatin C (*r* = 0.360, *p* = 0.007).

**Table 2.** Pathological characteristics of patients with severe DAIN.

	Total	Recovery group	Progression group	*p* Value
ATI (mild/moderate/severe), *n*	7/45/20	7/35/17	0/10/3	0.824
IFTA (mild/moderate/severe), *n*	19/7/1	14/1/1	5/6/0	<0.001
Interstitial inflammatory cell infiltration (mild/moderate/severe), *n*	21/33/18	21/27/11	0/6/7	0.002
Arterial intimal thickening, *n* (%)	38 (54.3%)	32 (52.4%)	6 (54.5%)	1.000

ATI: acute tubular injury; IFTA: interstitial fibrosis/tubular atrophy.

### Corticosteroids treatment of DAIN

Corticosteroids was prescribed to all the 72 patients, the time interval from symptom onset to treatment with corticosteroids was 16.8 ± 12.8 days. All of the 72 patients received intravenous methylprednisolone 40–80 mg/day during hospitalization, 5 of them received intravenously pulse methylprednisolone 500 mg/day for 3 days after renal biopsy. All the patients received oral prednisone after discharge, the initial dose of oral prednisone was 20–45 mg daily for 4 weeks, then tapered over 3–4 weeks. No other immunosuppressive medications were prescribed.

### Renal outcomes and risk factors

**Figure 2.**
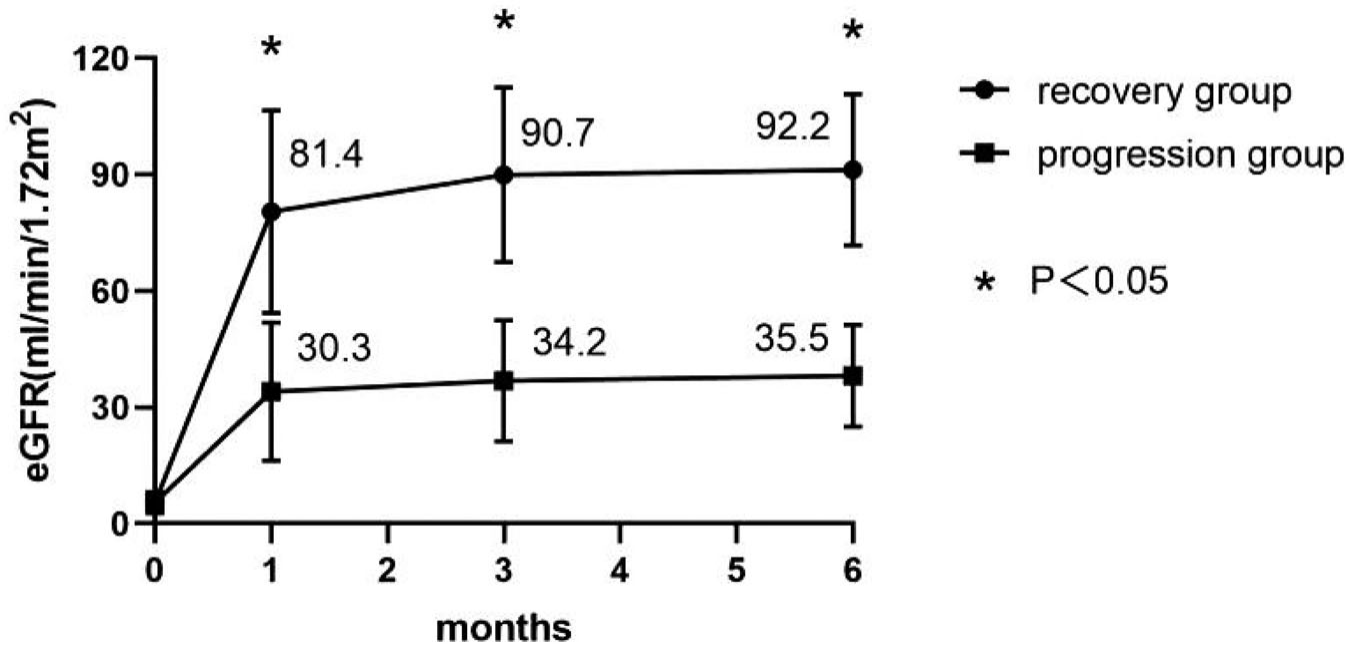
The restoration of renal dysfunction of patients from renal biopsy to the 6th month of follow-up in the recovery group and the progression group (* *p* < 0.05 versus progression group).

During at least 6 months of follow-up, 59 (81.90%) patients achieved complete recovery of renal function and 13 (18.10%) patients progressed to CKD. The eGFR increased more rapidly in the first month and kept stable during the 3–6 months of follow-ups, the eGFR was significantly higher in the recovery group than the progression group at months 1, 3, and 6 (Figure 2). In the recovery group, 70.8% patients achieved recovery of eGFR at month 1.

**Figure 3.**
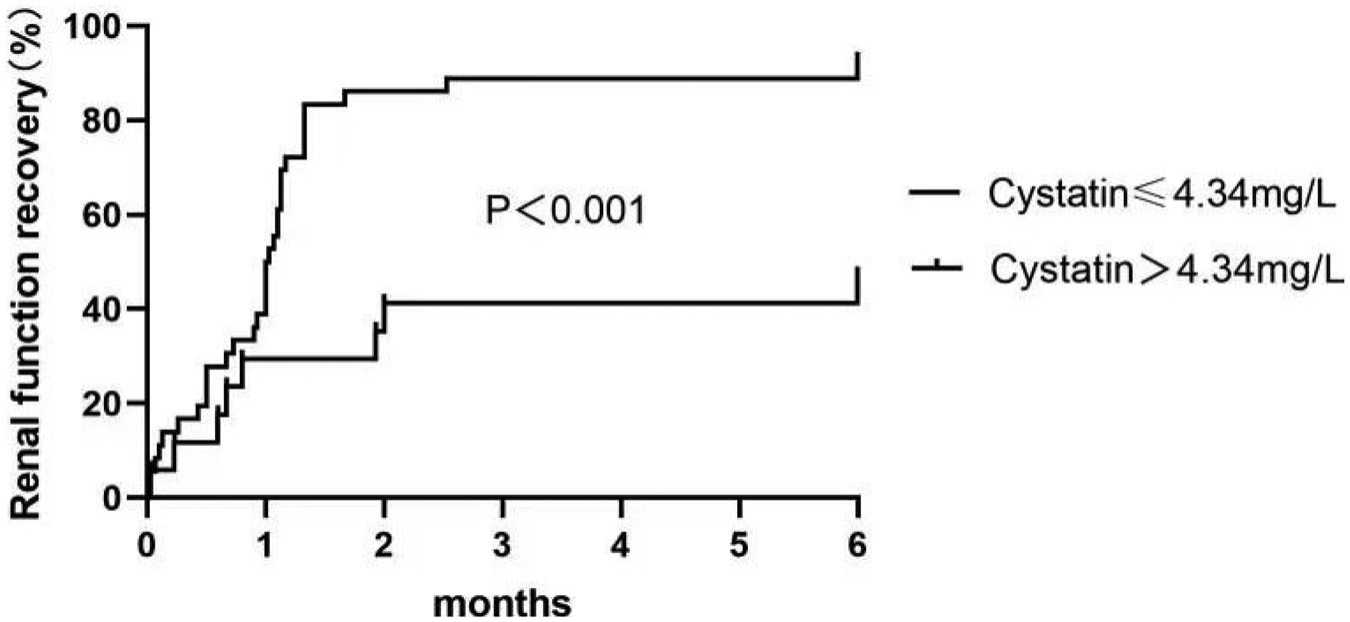
Kaplan–Meier survival curves shows the probability of renal recovery in patients with serum cystatin *C* > 4.34 mg/L and ≤4.34 mg/L.

**Figure 4.**
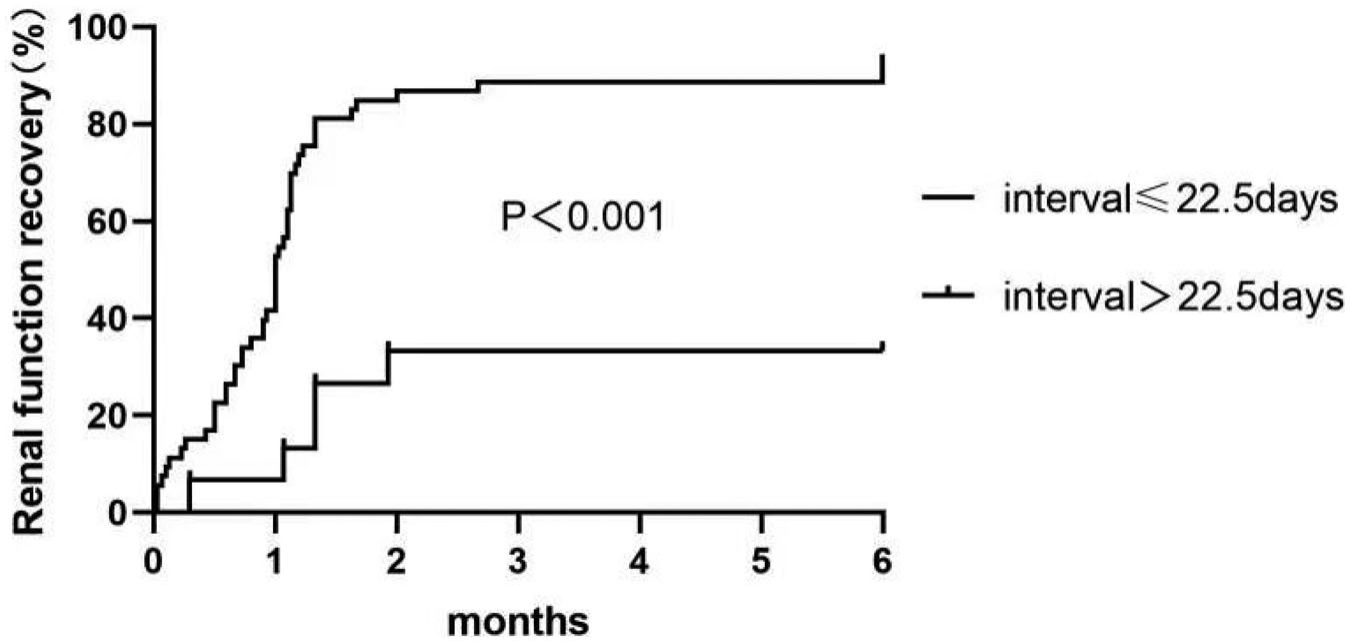
Kaplan–Meier survival curves shows the probability of renal function recovery in patients with interval to treatment with corticosteroids ≤ 22.5 days and > 22.5 days.

Univariate logistic analysis indicated that older age, higher serum peak cystatin C concentration, higher urine RBP concentration, longer interval from symptom onset to treatment with corticosteroids, higher score of IFTA, and more interstitial inflammatory cells infiltration were associated with the progression to CKD. The multivariable analysis indicated that higher serum peak cystatin C concentration (OR = 2.443, 95% CI 1.257, 4.746, *p* = 0.008) and longer interval to treatment with corticosteroids (OR = 1.183, 95% CI 1.035, 1.352, *p* = 0.014) were independently associated with the progression to CKD (Table 3). The cutoff value of cystatin C concentration was 4.34 mg/L, at which the sensitivity and specificity were 76.9% and 89.3%, respectively; the cutoff value of interval to treatment with corticosteroids was 22.5 days, at which the sensitivity and specificity were 81.8% and 79.5%, respectively. The proportion of renal function recovery decreased in patients with peak serum cystatin *C* > 4.34 mg/L or interval to corticosteroids treatment > 22.5 days with statistical significance (Figures 3 and 4).

**Table 3.** Logistic regression analysis for progression to CKD.

Variable	Univariate analysis		Multivariate analysis	
	0R (95% CI)	*p*-value	0R (95% CI)	*p* Value
Age (per 1 year increased)	1.073 (1.010, 1.139)	0.022		
Gender (M/F)	2.333 (0.681, 8.000)	0.178		
Hypertension, *n* (%)	0.533 (0.106, 2.686)	0.446		
Diabetes, *n* (%)	0.000 (0.000)	0.999		
Length of RRT-dependence (per 1 day increased)	0.990 (0.883, 1.110)	0.861		
Interval to treatment with steroids (per 1 day increased)	1.173 (1.070, 1.286)	0.001	1.183 (1.035, 1.352)	0.014
Cystatin C (per 1 mg/l increased)	2.789 (1.424, 5.462)	0.003	2.443 (1.257, 4.746)	0.008
Peak creatinine (per 1 mg/dl increased)	0.955 (0.770, 1.184)	0.672		
Proteinuria (per 1 g/24 h increased)	2.091 (0.848, 5.159)	0.109		
NAG (per 1 U/g*cr increased)	1.005 (0.992, 1.018)	0.489		
RBP (per 1 mg/l increased)	1.080 (1.027, 1.135)	0.003		
ATI (mild/moderate/severe), *n*	1.197 (0.629, 4.737)	0.733		
IFTA (mild/moderate/severe), *n*	0.826 (4.163, 98.346)	<0.001		
Interstitial inflammatory cell infiltration (mild/moderate/severe), *n*	4.637 (1.640, 13.113)	0.004		
Arterial intimal thickening, *n* (%)	0.675 (0.202, 2.254)	0.523		

## Discussion

In this retrospective observational study of patients with severe DAIN requiring RRT, all of the 72 patients weaned from RRT eventually. During at least 6 months of follow-up, majority of these patients achieved complete recovery of renal function with no albuminuria or hematuria, suggesting that patients with severe DAIN had a relatively good prognosis when they were identified and treated in time. However, there were still 13 patients progressed to CKD. Analyses indicated higher peak serum cystatin C concentration and longer interval to treatment with corticosteroids were independent risk factors for the progression to CKD, with the peak serum cystatin *C* > 4.34 mg/L or the interval to corticosteroids treatment > 22.5 days had the best predictive value of the progression to CKD.

Any drug can theoretically induce an episode of AIN. Types of suspicious drugs in our cohort consisted of antibiotics, NSAIDs, PPI, except four patients with traditional Chinese herbs, which consistent with most commonly implicated drug categories as reported. In our observation, all of these patients were treated with multiple drugs, so it was difficult to establish a causal relation to a specific drug.

The classic symptoms of drug allergy such as fever, skin rash, and peripheral eosinophilia were uncommon in our series, whereas majority of the patients had non-specific symptoms such as oliguria, loin pain, nausea, and gross hematuria, and a few of patients were even asymptomatic. The clinical scenario leading to a suspicion of DAIN would usually be a non-typical discomfort feeling after drugs exposure, progressive un-explained rise in serum creatinine levels with mild abnormal urinalysis. Hence, we suggest that more attention of DAIN should be paid, which may keep us to be more cautious and vigilant for drug prescription. In addition, it is important for early recognition of DAIN by continuous evaluating serum creatinine and urinalysis after drugs exposure.

This is the first study to investigate the prognosis of patients with severe DAIN requiring RRT and to identify the risk factors of progression to CKD. According to the results of previous studies in patients with AIN, renal function restored rapidly within the first 3 months post renal biopsy, poor recovery of renal function at 6 months was independent risk factor of worse long-term renal outcome [8,9], so we assessed the renal recovery status at 6 months after renal biopsy. Actually, the results also indicated that the eGFR increased significantly in the first month after renal biopsy and kept stable during the 3–6 months.

It has been attempted to find reliable serum biomarkers that predict renal recovery in AKI. Serum cystatin C concentration has long been known as a reliable biomarker for predicting AKI because it rises earlier, and less dependent on age, sex, race, diet, and muscle mass compared to serum creatinine. However, whether it is associated with renal function recovery had not been well established in previous studies. Zhang et al. [10] had reported that lower cystatin C concentration was an independent predictor of renal recovery in intensive care unit (ICU) patients with AKI of all-cause requiring continuous renal replacement therapy (CRRT), although the follow-up period was only 30 days. At the cutoff value of 3.13 mg/L, the sensitivity and specificity were 57.69% and 86.79%, respectively, indicating that patients with cystatin C value > 3.13 mg/L may have a poor renal outcome. Hu et al. [11] also reported cystatin C value independently predicted the risk of 2-year mortality and rehospitalization, as well as renal recovery failure in coronary care unit (CCU) patients with AKI. Our study provided more evidence to support the predictive performance of serum cystatin C concentration in identifying DAIN patients who are at risk for poor renal outcome with a cutoff value of 4.34 mg/L.

It is attractive for clinicians to consider the use of immunosuppressive agents for DAIN as it is an allergic inflammatory process. High-dose corticosteroids can rapidly alleviate T-cell infiltration and improve renal function. However, the benefit of corticosteroids treatment in AIN is not clear. There was no randomized controlled trial available to substantiate corticosteroids efficacy in patients with DAIN, evidence available to date was from small uncontrolled retrospective studies, which had shown conflicting results [5,8,12–15]. Most of these studies enrolled AIN patients of diverse etiology even though drug was the primary cause, and the decision of corticosteroids treatment was made by the physician, which induced selection bias in the observed association of corticosteroids with renal outcomes. In our center, administration of corticosteroids to the patients with severe DAIN without significant fibrosis in renal tissue is a standard of care, because we found drug withdrawal alone cannot take effects for normalizing renal function in a short time, and treatment without corticosteroids may leads to peril of gradual kidney fibrosis.

When focusing on corticosteroids treated patients with AIN, the available evidence seems to support a potential benefit of early corticosteroids administration on long-term recovery of kidney function in those without significant fibrosis in renal specimens. Gonza ´lez et al. [14] performed a multicenter retrospective study in 61 patients with biopsy-proven DAIN, 52 of whom were treated with corticosteroids. They found a significant correlation between the delay in steroids treatment and the final serum creatinine, and that an interval longer than 7 days between causative drug withdrawal and onset of corticosteroids treatment was the only clinical factor that significantly increased the risk of an incomplete recovery of renal function by multiple logistic regression analysis. In our present study, the cutoff value of corticosteroids initiation was 22.5 days. In most cases, a complete and rapid recovery of renal function generally occurred upon withdrawal of the causative drug and use of corticosteroids. Although the present study was also a retrospective observational study, we avoided the most important selection bias: the etiology of AIN, the degree of disease severity and choice of corticosteroids. In our study, we enrolled the patients with renal biopsy proven DAIN requiring RRT, and all the patients received treatment with corticosteroids. The results indicated longer interval to treatment with corticosteroids was an independent risk factor associated with the progression to CKD, which further emphasized the importance of early initiation of treatment with steroids in patients with severe DAIN.

This study also has several limitations. First, most of our cases have received multiple drugs before admitted to our center because of renal injury, so it was difficult to identify the causality of one or several definite culprit drugs and the duration time of relevant drugs. Second, because of the observational design, all the patients admitted to our center with AKI, the baseline serum creatinine levels were not available, and the initiation of RRT was according to criteria of azotemia and the decision of physician. Third, there were a relatively small number of patients enrolled and high rate of complete recovery, which may limit the statistical power to detect relevant factors associated with progression to CKD.

In summary, in this cohort of patients with severe DAIN requiring RRT, renal dysfunction was reversible in majority, even if RRT was instituted at presentation. Higher baseline serum cystatin C concentration was associated with poorer clinical outcome, early initiation of treatment with corticosteroids achieves a higher rate of renal recovery. Hence, early recognition of AKI by continuous monitoring serum creatinine and cystatin C, withdrawal of the causative drug, histologic confirmation, supportive treatment with RRT if necessary, early use of corticosteroids were of most importance for the prevention and treatment of severe DAIN.

## Data Availability

All the data supporting our findings is contained within the manuscript.
